# Correction: Promotional mechanism of enhanced denitration activity with Cu modification in a Ce/TiO_2_–ZrO_2_ catalyst for a low temperature NH_3_-SCR system

**DOI:** 10.1039/d1ra90179f

**Published:** 2022-01-10

**Authors:** Wei Zhang, Yunhao Tang, Wei Xiao, Min Ruan, Yanshan Yin, Quanbin Song, Kang Xie, Chuan Qin, Mengyao Dong, Yunhe Zhou, Jie Li

**Affiliations:** College of Energy and Power Engineering, Changsha University of Science & Technology Changsha 410114 China weizhang@csust.edu.cn; Key Laboratory of Renewable Energy Electric-Technology of Hunan Province Changsha 410114 China

## Abstract

Correction for ‘Promotional mechanism of enhanced denitration activity with Cu modification in a Ce/TiO_2_–ZrO_2_ catalyst for a low temperature NH_3_-SCR system’ by Wei Zhang *et al.*, *RSC Adv.*, 2022, **12**, 378–388, DOI: 10.1039/d1ra06325a

The authors regret the omission of a funding acknowledgement in the original article. This acknowledgement is given below.

We acknowledge the financial support from the Scientific Research Foundation of Hunan Educational Department, China (21A0216).

The Royal Society of Chemistry apologises for these errors and any consequent inconvenience to authors and readers.

## Supplementary Material

